# The Infectious Bursal Disease Virus RNA-Binding VP3 Polypeptide Inhibits PKR-Mediated Apoptosis

**DOI:** 10.1371/journal.pone.0046768

**Published:** 2012-10-09

**Authors:** Idoia Busnadiego, Ana M. Maestre, Dolores Rodríguez, José F. Rodríguez

**Affiliations:** Department of Molecular and Cellular Biology, Centro Nacional de Biotecnología-CSIC, Cantoblanco, Madrid, Spain; University of British Columbia, Canada

## Abstract

Infectious bursal disease virus (IBDV) is an avian pathogen responsible for an acute immunosuppressive disease that causes major losses to the poultry industry. Despite having a bipartite dsRNA genome, IBDV, as well as other members of the Birnaviridae family, possesses a single capsid layer formed by trimers of the VP2 capsid protein. The capsid encloses a ribonucleoprotein complex formed by the genome associated to the RNA-dependent RNA polymerase and the RNA-binding polypeptide VP3. A previous report evidenced that expression of the mature VP2 IBDV capsid polypeptide triggers a swift programmed cell death response in a wide variety of cell lines. The mechanism(s) underlying this effect remained unknown. Here, we show that VP2 expression in HeLa cells activates the double-stranded RNA (dsRNA)-dependent protein kinase (PKR), which in turn triggers the phosphorylation of the eukaryotic initiation factor 2α (eIF2α). This results in a strong blockade of protein synthesis and the activation of an apoptotic response which is efficiently blocked by coexpression of a dominant negative PKR polypeptide. Our results demonstrate that coexpression of the VP3 polypeptide precludes phosphorylation of both PKR and eIF2α and the onset of programmed cell death induced by VP2 expression. A mutation blocking the capacity of VP3 to bind dsRNA also abolishes its capacity to prevent PKR activation and apoptosis. Further experiments showed that VP3 functionally replaces the host-range vaccinia virus (VACV) E3 protein, thus allowing the E3 deficient VACV deletion mutant WRΔE3L to grow in non-permissive cell lines. According to results presented here, VP3 can be categorized along with other well characterized proteins such us VACV E3, avian reovirus sigmaA, and influenza virus NS1 as a virus-encoded dsRNA-binding polypeptide with antiapoptotic properties. Our results suggest that VP3 plays a central role in ensuring the viability of the IBDV replication cycle by preventing programmed cell death.

## Introduction

Virus replication entails a complex set of interactions between the host cell machinery and viral products that has the potential to alter cell homeostasis. Indeed, every step in virus replication is susceptible to activate proapoptotic signaling. Programmed cell death (PCD) is a mechanism designed to eliminate superfluous cells, but it also acts as a first line of defense to halt virus replication. Although as a general rule PCD is very efficient at lessening the production of viral progenies, in some cases it might also contribute to virus dissemination and pathogenicity. In accordance to its importance, viruses incorporate a wide variety of molecular devices intended to counteract and/or manipulate the host's PCD response [1].

Apoptosis is induced by the activation of a family of cysteine proteases generically known as caspases. Caspase activation is triggered by two different but interrelated pathways [2]. The intrinsic pathway, also known as mitochondrial pathway, is set off following the detection of different types of cellular stress by specific sensor proteins belonging to the BH3-only member of the Bcl-2 family. This results in the formation of the apoptosome that prompts the activation of effector proteases, namely caspase-3 and -7 [3]. The extrinsic pathway is activated by ligation of receptors containing death domains located at the cell membrane. This leads to the formation of the death-induced signaling complex (DISC) that triggers the activation of the initiator caspase-8 [4]. In some cell types, caspase-8 activation triggers a direct activation of the caspase-3 and -7 effectors, whilst in others it requires the amplification of death signals through stimulation of the mitochondrial pathway via the Bid sensor protein, also a member of the Bcl-2 family [5].

The infectious bursal disease virus (IBDV) is the best characterized member of the *Birnaviridae* family that groups naked icosahedral viruses with bi-segmented double-stranded RNA (dsRNA) genomes [6]. IBDV infects different bird species and causes an acute immunosuppressive disease, known as IBD or Gumboro disease, that affects domestic chickens (*Gallus gallus*) and it is responsible for major economic losses to the poultry industry world-wide [7]. Chickens are highly susceptible to the infection between 3 and 6 weeks after hatching. The consensus is that, following the ingestion of virus-contaminated stuff, virus particles are taken up by resident gut macrophages, and then transported to other tissues. After reaching the Fabricius bursa (FB), the main IBDV-target organ, the virus actively replicates in IgM-bearing B lymphocytes [8]. Infection results in a rapid depletion of the bursal B-cell population but other secondary lymphoid organs, such as the spleen, thymus and cecal tonsils, also become depleted [9]. The destruction of lymphocyte populations associated to the infection causes immune suppression and hampers the immunological maturation of infected birds [10].

IBDV possess a single capsid (T = 13L symmetry) formed by 260 trimers of the capsid polypeptide (VP2) [11]. The inner capsid space is occupied by a ribonucleoprotein complex (RNP) formed by the dsRNA genome wrapped up by the VP3 polypeptide and covalently linked to the VPg form of the VP1 RNA-dependent RNA polymerase (RdRp), and by “free” VP1 molecules thought to act both as primer and polymerase during RNA transcription [12], [13].

The IBDV genome is formed by two dsRNA segments (A and B) encoding a total of five mature proteins. Segment A (3.2 kb) harbors two largely overlapped open reading frames (ORF) (ORF A1 and A2, respectively). Segment A transcription generates a single bicistronic mRNA. ORF A1 encodes VP5, a small (17-kDa), non-structural (NS) polypeptide, dispensable for virus replication in cell culture, that has been shown to be directly associated to virus dissemination and virulence [14]–[17]. ORF A2 encodes a large (107 kDa) polyprotein that undergoes a co-translational self-proteolytic processing that releases three polypeptides, namely the precursor of the capsid protein (pVP2, 54 kDa), the protease (VP4, 25 kDa), and the multifunctional polypeptide VP3 (28 kDa) [18]. The C-terminal pVP2 domain is subsequently cleaved at three secondary targets by VP4, and finally processed by VP2 itself to render the mature VP2 polypeptide (38 kDa) [19], [20]. Segment B (2.8 kb) contains a single ORF (ORF B) encoding the RdRp VP1 (97 kDa) polypeptide [21].

Studies carried out in IBDV-infected birds documented the presence of apoptotic signs in infected bursal lymphocytes as well as in cells lacking detectable levels of IBDV-encoded polypeptides. This observation suggests that in vivo IBDV indirectly induces apoptosis in uninfected bystander cells, and this might contribute to the fast depletion of B lymphocytes and the destruction of the FB and immunosuppression [22]. Characterization of the IBDV replication cycle in chicken embryo fibroblasts (CEF) evidenced that, in this cell type, IBDV triggers a PCD response during the late phase of virus replication, after the assembly of the virus progeny. Two IBDV-encoded polypeptides, namely VP5 and VP2, have been related to the induction and control of IBDV-induced apoptotic response. The role of VP5 remains unclear. VP5 was first described as playing an anti-apoptotic role during the initial stages of IBDV infection [23]. Two recent reports have described the ability of VP5 to interact with the voltage-dependent anion channel 2 polypeptide (VDAC2) [24] and with p85α subunit of the phosphatidylinositol 3-kinase (PI3K) [25], respectively. According to these reports, the interaction of VP5 with VDAC2 promotes PCD and restricts virus replication, whilst its interaction with the PI3K p85α subunit suppresses premature apoptosis and enhances virus progeny yields. Regarding VP2, it has been shown that cells expressing the VP2 ORF in the absence of other virus genes using either vaccinia virus (VACV) or plasmid-based eukaryotic expression vectors, undergo a vigorous shut off of protein synthesis followed by PCD in a variety of cell lines [26]. This observation contrasts with the finding that expression of the complete IBDV polyprotein gene, that includes the VP2 coding region, leads to an efficient accumulation of polyprotein-derived products and the assembly of virus-like particles in the absence of a detectable PCD response [27]. The molecular mechanisms responsible for both the PCD induction observed in VP2-expressing cells and the differential outcome observed in cells expressing VP2 and the polyprotein genes remained unknown. Results described in this report demonstrate that VP2 expression induces the activation of the dsRNA-dependent protein kinase (PKR)-mediated PCD response, and that this effect is efficiently blocked by coexpression of the IBDV RNA binding VP3 polypeptide.

VACV encodes a battery of polypeptides involved in the control of antiviral innate host responses [28], [29]. One of these proteins, E3, is a dsRNA-binding polypeptide responsible for the inhibition of interferon (IFN)-induced pathways. E3 sequesters dsRNA molecules generated during the VACV replication process and prevents the activation of two IFN-inducible enzymes: PKR, responsible for the phosphorylation of the alpha subunit of the eukaryotic translation initiation factor 2 (eIF2α) and the subsequent shut off of protein synthesis; and 2′-5′-oligoadenylate synthetase (2′-5′OAS) that triggers the activation of RNase L which in turn degrades both cellular and viral RNAs [30]. Elimination of the E3L gene from the VACV genome causes a major restriction in the range of cell lines supporting a productive VACV infection [31]. Here we show that VP3 functionally replaces in vitro the VACV dsRNA-binding E3 protein. Data described here suggest that the VP3 polypeptide plays an important role in counteracting IBDV-induced innate antiviral host cell responses.

## Materials and Methods

### Cells, viruses, infections, and transfections

HeLa (human epithelial cervical cancer cells, ATCC number CCL-2TM), DF-1 (spontaneously transformed chicken embryo fibroblasts, ATCC number CRL-12203), and BSC40 (African green monkey kidney cells, ATCC number CRL-2761) were grown in Dulbecco's modified minimal essential medium (DMEM) supplemented with penicillin (100 U/ml), streptomycin (100 mg/ml) and 10% fetal calf serum (FCS) (Sigma). VACV VT7LacOI (referred here as VT7 for simplicity, was kindly provided by B. Moss. National Institutes of Health, Bethesda, Maryland, USA) [32], VT7/VP2 [26], VT7/VP3 [26], VT7/VP3P1, the Western reserve strain (WR) [33], and WR/PKR-NP [34] (kindly provided by M. Esteban. Centro Nacional de Biotecnologia. CSIC. Madrid. Spain) were grown and titrated in BSC40 cells. VACV WRΔE3L [35] (kindly provided by B. Jacobs, Arizona State University, Tempe, Arizona, USA) and WRΔE3L/VP3 were grown and titrated in DF-1 cells. All VACV were purified through two consecutive 45% (w/v) sucrose cushions, and titrated in triplicate by plaque assay. Infections were performed on preconfluent (≈75%) cell monolayers. Cultures were mock-infected or infected with the different viruses diluted in DMEM to the indicated multiplicity of infection (MOI). After 1 h of adsorption at 37°C the medium was removed and replaced with fresh DMEM supplemented with 2% FCS. The expression of isopropyl β-D-thiogalactosidase (IPTG)-inducible genes was triggered by adding IPTG (Apollo Scientific), 1 mM final concentration, to cell culture medium immediately after virus adsorption. Infected cells were incubated at 37°C until the specified times post-infection (p.i.). Transfections were performed with lipofectamine 2000 reagent (Invitrogen) on pre-confluent (80%) HeLa cell monolayers using a plasmid concentration of 100 ng per 10^5^ cells.

### Construction of recombinant VACV

To construct VT7/VP3P1, a DNA fragment of 789 bp containing the VP3MutPatch1 mutant version of the VP3 ORF, containing four amino acid substitutions (K99D, R102D, K105D and K106D), flanked by NdeI and BamHI restriction sites, was generated by PCR using plasmid pFBhisVP3Patch1 (unpublished results) as template and the primers 5′GCGCCATATGGCTGCATCAGAGTTCAAAGAG and 5′GCGCGGATCCTCACTCAAGGTCCTCATCAGAG. The resulting PCR product was purified, digested with NdeI and BamHI and ligated to pVOTE.2 [32] previously digested with the same restriction enzymes. The resulting plasmid vector, pVOTE/VP3P1, was subjected to nucleotide sequencing to assess the correctness of the inserted sequence, and then used to generate the VACV VT7/VP3P1 via homologous recombination between the plasmid vector and the genome of the parental VT7 VACV. For this, BSC40 cells were infected with VT7 [32] and transfected with pVOTE/VP3P1. Selection and amplification of VT7/VP3P1 were carried out in BSC40 cells following a previously described protocol [32].

Construction of WRΔE3L/VP3 was initiated by producing a PCR-derived DNA fragment of 789 bp containing the VP3 ORF flanked by KpnI and BamHI restriction sites. The PCR reaction was carried out using the previously described pcDNA3-POLY plasmid [19] as template and oligonucleotides 5′GCGCGGTACCATGGCTGCATCAGAGTTCAAAGAG and 5′GCGCGGATCCTCACTCAAGGTCCTCATCAGAG as primers. After purification, the DNA fragment was subjected to restriction with KpnI and BamHI and ligated to the transfer plasmid vector pJR101 [36]. The resulting vector, pJR101/VP3, contains an insertion cassette formed by the VP3 ORF placed under the transcriptional control of the synthetic early/late Pse/l VACV promoter, and the *E. coli* β-glucoronidase selection marker gene controlled by the P7.5 early/late promoter. The insertion cassette is flanked by sequences corresponding to the VACV hemagglutinin (HA) coding gene (A56R gene). pJR101/VP3 was subjected to nucleotide sequencing to assess the correctness of the inserted sequence and then used to transfect DF-1 cell monolayers previously infected with the parental virus WRΔE3L. The selection and amplification of WRΔE3L/VP3 was performed in DF-1 cells as previously described [36].

### Construction of pcDNA-VP3

A DNA fragment corresponding to the VP3 coding region was generated by PCR from pVOTE.1/VP3 [26] using the primers 5′-CGCGAAGCTTATGGGTTTCCCTCACAATCCACGC and 5′-GCGCGGATCCTCACTCAAGGTCCTCATCAGAGAC. The resulting fragment contains an artificial ATG codon to allow for initiation of translation. The DNA fragment was purified, restricted with HindIII and BamHI and cloned into pcDNA3 (Invitrogen) previously digested with the same enzymes. The resulting plasmid, pcDNA-VP3, was subjected to nucleotide sequence analysis to assess the correctness of the cloned sequence.

### Determination of caspase 3/7 activation

Determinations were carried out using the Caspase-Glo 3/7 assay kit (Promega) following the protocol recommended by the supplier. Briefly, HeLa cell monolayers grown in 96 well plates were infected at the indicated MOI. At the specified times p.i., 100 µl of Caspase-Glo 3/7 reagent was added to the wells under study. Plates were gently shaken and then incubated in the dark at 20°C for 60 min before recording the luciferase activity using an Orion microplate luminometer (Berthold technologies).

### Autoradiography and Western blot analysis

For metabolic labeling cell monolayers were washed twice with methionine-free DMEM. Thereafter, cultures were incubated for 30 min with 100 µCi/ml of [^35^S]methionine, washed twice with PBS, and resuspended in Laemmli's sample buffer (62.5 mM Tris-HCl [pH 6.8]; 2% sodium dodecyl sulfate [SDS]; 0.25% bromophenol blue; 5% glycerol; and 5% β-mercaptoethanol). Protein samples were subjected to 12% SDS-polyacrylamide gel electrophoresis (SDS-PAGE), fixed, and dried. Radioactive signals were detected with a Storm gel imaging system (Molecular Dynamics).

Samples used for Western blot (WB) analysis were prepared by removing media from cell monolayers and resuspending the cells in iced-chilled disruption buffer (0.5% Triton X-100; 50 mM KCl; 50 mM NaCl; 20 mM Tris-HCl [pH 7.5]; 1 mM EDTA, 10% glycerol; Complete protease inhibitor cocktail [Roche]; 25 mM β-glycerophosphate; 1 mM Na_3_VO_4_). Cell lysates were mixed (v/v) with 2× Laemmli's sample buffer. Electrophoreses were performed in 12% SDS-PAGE, followed by electroblotting onto nitrocellulose membranes. Immunoblots were blocked for 1 h in PBS containing 0.05% Tween 20 (PBST) and 5% non-fat dry milk, washed in PBST, and incubated at 4°C overnight with the different primary antibodies in PBST containing 1% non-fat dry milk. Antibodies used in this study were rabbit polyclonal sera specific for IBDV VP2 [27] and VP3 [27], VACV D13 [37], pT451 PKR (Invitrogen), total eIF2α (Santa Cruz Biotechnology), pS52 eIF2α (Invitrogen), respectively, and mouse monoclonals specific for VACV E3 (a generous gift from B. Jacobs, Arizona State University, Tempe, Arizona, USA), β-actin (Sigma), and total PKR (Santa Cruz Biotechnology). After incubation with primary antibodies, membranes were incubated with either goat anti-rabbit IgG-Peroxidase conjugate (Sigma) or goat anti-mouse IgG-Peroxidase conjugate (Sigma), and immunoreactive bands detected by enhanced chemiluminescence reaction (GE Healthcare). Blocking and primary antibody incubation for total eIF-2α, pS52 eIF-2α, total PKR, and pT451 PKR were performed according to instructions provided by the manufacturer.

## Results

### VP2 expression leads to PKR and eIF2α phosphorylation

**Figure 1:**
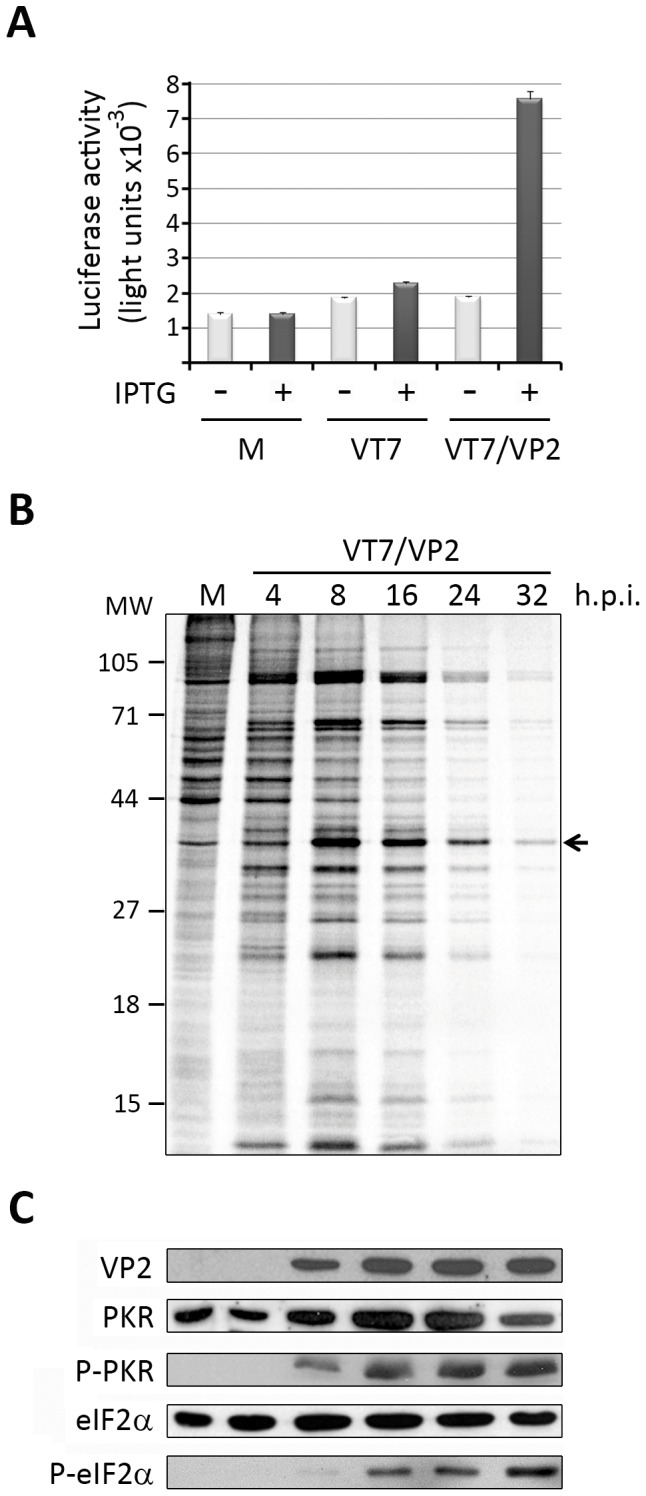
Charaterization of cell responses triggered by VP2 expression in HeLa cells. A. Induction of apoptosis. Cell monolayers were mock-infected (M) or infected (MOI 2 PFU/cell) with the VT7 or VT7/VP2 viruses. After infection, cultures were maintained in the absence (−) or presence (+) of the inducer IPTG. Apoptosis was measured using the Caspase-Glo 3/7 assay kit at 24 h.p.i. Each determination was carried out in triplicate. Presented data correspond to the mean ± the standard deviation of three independent experiments. **B. Host cell protein synthesis.** The panel shows an autoradiography corresponding to a time-course analysis carried out in IPTG-induced VT7/VP2-infected HeLa cells. At the indicated times (h.p.i.) cells were metabolically labeled with [^35^S]methionine. A mock-infected culture (M) was used as an internal control. Cell extracts were subjected to SDS-PAGE and autoradiography. The position of molecular mass markers is indicated (MW). The band corresponding to the VP2 polypeptide is indicated by an arrow. **C. PKR and eIF2α phosphorylation.** Total cell extracts from mock- or VT7/VP2-infected cells collected at the times p.i. (h.p.i.) indicated in panel B were subjected to SDS-PAGE, transferred to nitrocellulose, and immunoblotted with serum anti-VP2, -PKR, -pT451 PKR, -eIF2α, or -pS52 eIF2α. The WB corresponding to eIF2α was used as protein loading control.

We have previously shown that VP2 expression induces a potent shut off of protein synthesis that is followed by a PCD response [26]. The molecular basis underlying this effect remained unknown. To gain insight about this phenomenon we used a previously described recombinant VACV, VT7/VP2, that expresses the VP2 polypeptide upon addition to the cell culture medium of the inducer IPTG [32]. HeLa cell monolayers were infected with either VT7, the parental virus used to generate VT7/VP2, or with VT7/VP2 at a MOI of 2 plaque-forming units per cell (PFU/cell) and maintained in medium supplemented or not with IPTG. At 24 h p.i. the apoptotic response induced by these two viruses was measured by analyzing the activation of the effector caspases 3 and 7 using the caspase-Glo 3/7 assay kit that is based on the release of luciferase substrate mediated by the specific activitity of caspases 3 and 7. Basal caspase activation levels were determined using mock-infected cells maintained in the absence of IPTG. As shown in Fig. 1A, in cultures infected with the parental VT7 VACV the addition of IPTG to the cell medium caused a moderate increase (less that 1,5 fold) on the caspase activation levels. This observation sharply contrasts with data gathered with cultures infected with VT7/VP2. In this case, addition of IPTG triggered a significant increase (≈4 fold) on the caspase activation levels with respect to those observed in its corresponding IPTG-untreated control.

To analyze in more detail the effect of VP2 expression on cell fate, two sets of HeLa cell cultures were infected with this virus (2 PFU/cell) and maintained in medium supplemented with IPTG. IPTG-treated uninfected cells were used as a control for this experiment. The first culture set was used to assess the kinetics of protein synthesis. For this, at different times p.i., ranging from 4 to 32 h, cells were metabolically labeled with [^35^S]methionine for 30 min, and the corresponding samples subjected to SDS-PAGE followed by autoradiography (Fig. 1B). The second culture set was used to assess the status of selected polypeptides by WB analysis (Fig. 1C).

In agreement to previously reported data [26], we observed that cells expressing VP2 undergo a potent shut off of protein synthesis evidenced by the steady reduction of [^35^S]methionine incorporation detected in samples collected from 16 h.p.i. onwards (Fig. 1B). The WB analysis performed with the VP2-specific serum shows that VP2 accumulation is already detectable at 8 h.p.i., and reaches its maximum level at 16 h.p.i. (Fig. 1C). In addition to the described biochemical changes, cells expressing VP2 exhibited noticeable morphological alterations, e.g. cell shrinkage and membrane blebbing, typically found in apoptotic cells (data not shown).

One of the most common causes for the inhibition of protein synthesis in virus-infected cells is the phosphorylation of eIF2α [38]. Hence, we analyzed the extent of eIF2α phosphorylation in VP2-expressing cells. As shown in Fig. 1C, whilst the level of total eIF2α remains roughly constant throughout the duration of the experiment, the presence of phosphorylated eIF2α (P-eIF2α), first noticeable as a very faint band in samples collected at 8 h.p.i., increases with time, thus somehow matching the VP2 expression profile. It has been shown that eIF2α can be phosphorylated by four mammalian serine-threonine protein kinases, namely PKR, general non-derepressible 2 kinase (GCN2), PKR-like endoplasmic reticulum kinase (PERK), and hemin-regulated inhibitor of translation (HRI), following diverse stress conditions [39]–[42]. In particular, PKR has been shown to be a chief contender in the defensive host cell responses against a wide variety of viruses [43]. Consequently, we decided to analyze whether the presence of phosphorylated eIF2α (P-eIF2α) in cells expressing VP2 might be paralleled by the accumulation of phosphorylated PKR (P-PKR). Results presented in Fig. 1C show that, in line to what had been observed with the eIF2α polypeptide, the presence of P-PKR, already detectable in samples harvested at 8 h.p.i., reaches a maximum intensity level from 16 to 32 h.p.i. As expected, no significant variations were observed in the total content of PKR throughout the duration of the infection. The results of this analysis indicate that the blockade of protein synthesis and the PCD response observed in cells expressing the VP2 polypeptide could be initially triggered by phosphorylation of PKR.

### Expression of a dominant-negative version of PKR prevents VP2-induced protein synthesis arrest and PCD

Results described above strongly suggested that the protein synthesis blockade and the PCD response observed in cells expressing VP2 might be the result of eIF2α phosphorylation triggered by activated PKR. To test this hypothesis we took advantage of the previously described recombinant WR/PKR-NP generated using the WR strain of VACV as parental virus [34]. Upon IPTG addition to the cell medium, WR/PKR-NP expresses a dominant negative mutant form of the PKR polypeptide containing a single amino acid substitution (K269R) that is unable to phosphorylate eIF2α. To examine the role of PKR phosphorylation in the arrest of protein synthesis and the PCD response observed in VP2 expressing cells, HeLa monolayers were coinfected either with VT7/VP2+WR or VT7/VP2+WR/PKR-NP and maintained in the presence or absence of IPTG for 24 h. These cultures were used to assess the level of caspase 3/7 activation, the status of protein synthesis, and the comparative accumulation of relevant polypeptides.

**Figure 2:**
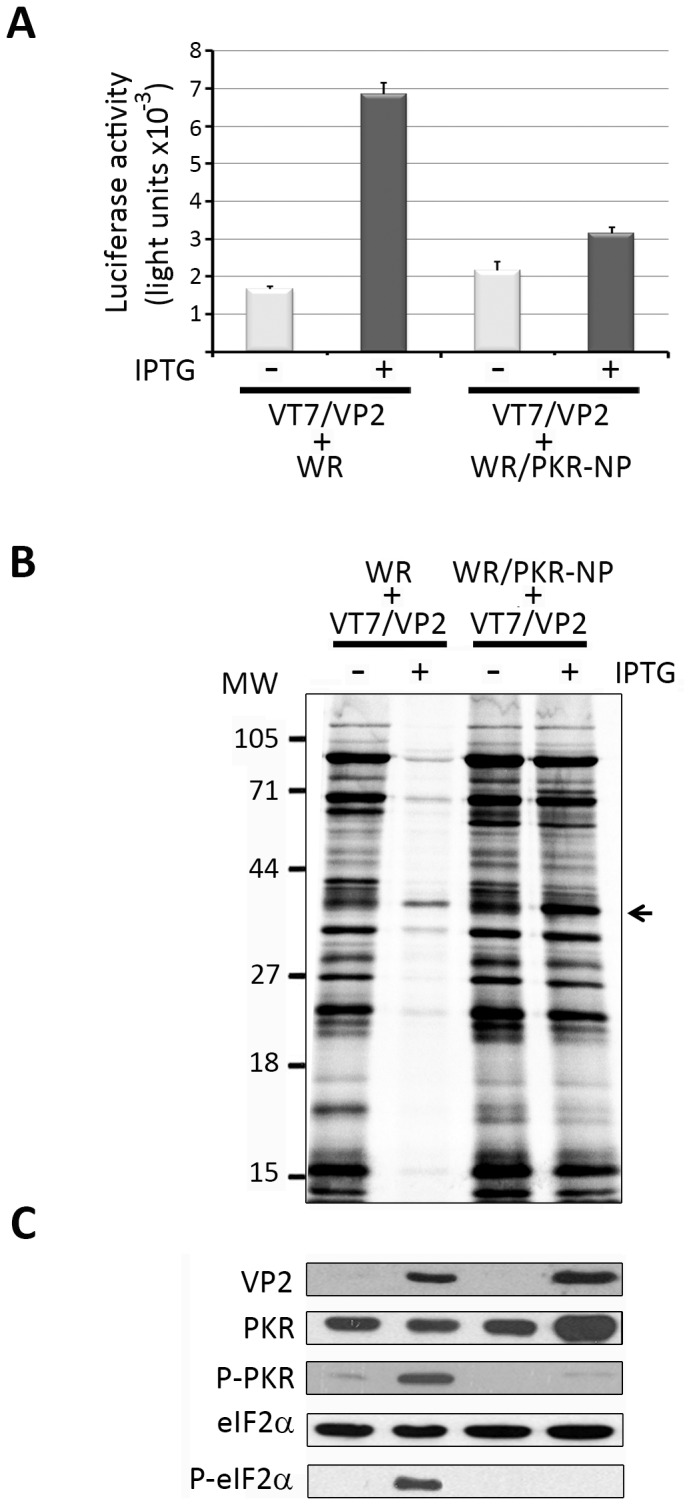
Effect of the expression of a dominant negative PKR on VP2-induced cell responses. A. Induction of apoptosis. Cell monolayers were coinfected with VT7/VP2+WR or VT7/VP2+WR/PKR-NP (2 PFU/cell of each virus) and maintained in the absence (−) or presence (+) of the inducer IPTG. Apoptosis was measured using the Caspase-Glo 3/7 assay kit at 24 h.p.i. Each determination was carried in triplicate. Presented data correspond to the mean ± the standard deviation of three independent experiments. **B. Host cell protein synthesis.** The panel shows an autoradiography corresponding to the protein synthesis profiles obtained from cells infected with VT7/VP2+WR or VT7/VP2+WR/PKR-NP that were maintained in the absence (−) or presence (+) of IPTG and metabolically labeled with [^35^S]methionine at 24 h.p.i. Cell extracts were subjected to SDS-PAGE and autoradiography. The position of molecular mass markers is indicated (MW). The radioactive band corresponding to the VP2 polypeptide is indicated by an arrow. **C. PKR and eIF2α phosphorylation.** Total extracts from cells infected with VT7/VP2+WR or VT7/VP2+WR/PKR-NP, maintained in the absence (−) or presence (+) of IPTG, were collected at for 24 h.p.i. Extracts were subjected to SDS-PAGE, transferred to nitrocellulose, and immunoblotted with serum anti-VP2, -PKR, -pT451 PKR, -eIF2α, or -pS52 eIF2α. The WB corresponding to eIF2α was used as protein loading control.

As shown in Fig. 2A, coinfection with WR does not significantly alter the level of caspase 3/7 activation induced by VP2 expression compared with that observed in cells infected with VT7/VP2 alone (Fig. 1A). In contrast, coinfection with WR/PKR-NP results in a major reduction of caspase 3/7 activation (Fig. 2A). A similar effect was observed concerning the impact of VP2 on protein synthesis. Whilst coinfection with WR does not cause a noticeable effect on the shut off of protein synthesis observed in cells expressing VP2, cells coinfected with VT7/VP2+WR/PKR-NP show protein synthesis levels similar to those observed in uninduced cells infected with VT7/VP2 (Fig. 2B). The arrest of protein synthesis observed in samples corresponding to cells coinfected with VT7/VP2+WR and maintained in the presence of IPTG was accompanied by the presence of VP2 and a conspicuous accumulation of P-PKR and P-eIF2α (Fig. 2C). IPTG-induced cultures coinfected with VT7/VP2+WR/PKR-NP accumulated a comparatively higher amount of the VP2 polypeptide but showed a barely detectable P-PKR level. Additionally, the presence of P-eIF2α was not detectable in these cultures. As expected, whilst the amount of total eIF2α was similar in all analyzed cell extracts, the amount of total PKR was noticeable enriched in extracts from IPTG-induced cells coinfected with VT7/VP2+WR/PKR-NP (Fig. 2C).

Results presented in this section demonstrate that the arrest of protein synthesis and the PCD response observed in HeLa cells expressing the VP2 polypeptide are efficiently counteracted by the expression of a dominant negative form of PKR. The presence of this polypeptide largely precludes the VP2-induced phosphorylation of PKR and completely prevents the subsequent phosphorylation of eIF2α, thus pointing the PKR phosphorylation observed en VP2-expressing cells as the leading cause of the activation of a PCD response.

### Coexpression of the IBDV VP3 polypeptide prevents VP2-induced protein synthesis arrest and PCD

Data described above demonstrates that VP2 expression triggers PKR activation and the phosphorylation of eIF2α, hence leading to a blockade of protein synthesis and PCD. This observation sharply contrasts with results obtained when expressing the IBDV polyprotein using the same expression system. The expression of the polyprotein gene using VT7/Poly, a recombinant VACV generated by inserting the polyprotein gene into the genome of the VT7 vector, leads to a rather efficient accumulation of polyprotein-derived polypeptides in the absence of a PCD response [27]. This observation suggested the possibility that the effects prompted by VP2 expression might be counteracted by other polyprotein-derived product. Likewise other well characterized virus-encoded proteins capable of preventing PKR activation, e.g. VACV E3 [34], avian reovirus sigmaA [44] or influenza virus NS1 [45], VP3 efficiently binds dsRNA in a non-specific manner [46].

In view of the results described above, we considered the VP3 polypeptide as a likely candidate to prevent VP2-mediated PKR activation. To analyze this hypothesis we used a previously described recombinant VACV, VT7/VP3, that expresses the IBDV VP3 polypeptide in the presence of IPTG [26]. As control for the experiments described below, we used the VT7 virus, the VACV employed as a parental virus for the generation of the VT7/VP2 and VT7/VP3 recombinant viruses. VT7 carries all the foreign genetic regulatory elements present in both VT7/VP2 and VT7/VP3. A series of single and double infections were carried out with VT7, VT7/VP2 and VT7/VP3 viruses. Infections were performed with 2 PFU/cell of each virus, and thereafter cultures were maintained in the absence or presence of IPTG for 24 h. Three cell sets were used in order to assess the level of caspase 3/7 activation, protein synthesis, and the comparative accumulation of relevant polypeptides.

**Figure 3:**
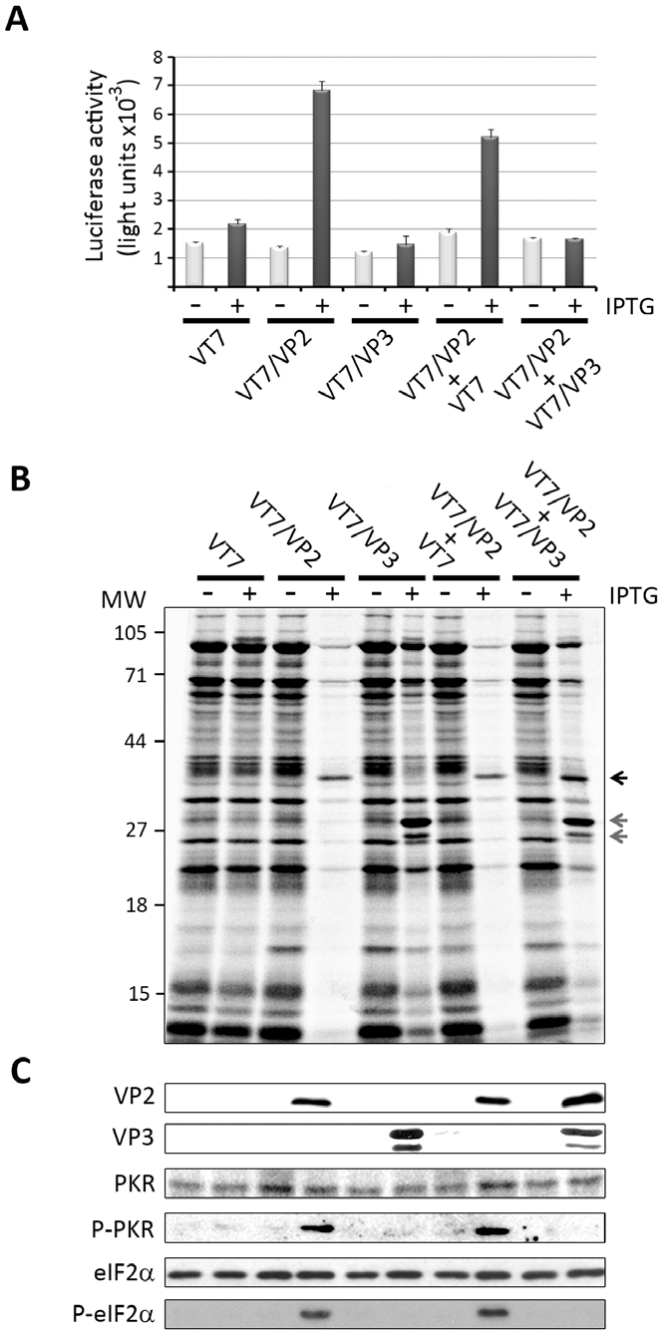
The expression of the IBDV VP3 polypeptide counteracts VP2-induced cell responses. A. Induction of apoptosis. Cell monolayers were infected with VT7, VT7/VP2 or VT7/VP3, or coinfected with VT7+VT7/VP2 or VT7/VP3+VT7/VP2 (2 PFU/cell of each virus) and maintained in the absence (−) or presence (+) of the inducer IPTG. Apoptosis was measured using the Caspase-Glo 3/7 assay kit at 24 h.p.i. Each determination was carried in triplicate. Presented data correspond to the mean ± the standard deviation of three independent experiments. **B. Host cell protein synthesis.** The panel shows an autoradiography corresponding the protein synthesis profiles obtained from cells infected with VT7, VT7/VP2 or VT7/VP3, or coinfected with VT7+VT7/VP2 or VT7/VP3+VT7/VP2. Cultures were maintained in the absence (−) or presence (+) of IPTG. At 24 h.p.i. cells were metabolically labeled with [^35^S]methionine. Cell extracts were subjected to SDS-PAGE and autoradiography. The position of molecular mass markers is indicated (MW). Arrows indicate the position of bands corresponding to the VP2 polypeptide (black) and VP3 (grey). **C. PKR and eIF2α phosphorylation.** Total extracts from cells infected with VT7, VT7/VP2 or VT7/VP3, or coinfected with VT7+VT7/VP2 or VT7/VP3+VT7/VP2, maintained in the absence (−) or presence (+) of IPTG, were harvested at 24 h.p.i. Extracts were subjected to SDS-PAGE, transferred to nitrocellulose, and immunoblotted with serum anti-VP2, -VP3, -PKR, -pT451 PKR, -eIF2α, or -pS52 eIF2α. The WB corresponding to eIF2α was used as protein loading control.

As expected from data presented above, the addition of IPTG to cultures infected with VT7/VP2 either alone or coinfected with the parental VT7 virus triggered a notorious apoptotic response (Fig. 3A). However, the level of caspase 3/7 activation detected in ITPG-induced cells coinfected with VT7/VP2 and VT7/VP3 were similar to those found in uninduced cultures. The activation levels found in cells coinfected with VT7/VP2 and VT7/VP3 were akin to those found after infection with either the parental VT7 or the VT7/VP3 viruses. In line with these observations, a clear arrest of protein synthesis was detected exclusively in IPTG-induced cultures infected with VT7/VP2 alone or coinfected with VT7 (Fig. 3B). As expected, the shut off of protein synthesis was accompanied by a prominent accumulation of both P-PKR and P-eIF2α (Fig. 3C). In a sharp contrast, cells infected with VT7/VP2+VT7/VP3, simultaneously expressing the VP2 and VP3 polypeptides, exhibited similar protein synthesis rates to those found in IPTG-induced cells infected with either the parental virus VT7 or the recombinant VT7/VP3 (Fig. 3B). Consistent with the preservation of normal levels of protein synthesis, extracts from cells simultaneously expressing the VP2 and VP3 polypeptides (Fig. 3B, C) contained undetectable levels of P-PKR and P-eIF2α (Fig. 3C). As shown before [26], VP3 overexpression gives rise to accumulation of a protein doublet formed by a major band of the expected SDS-PAGE mobility along with a faint band with a slightly faster mobility.

**Figure 4:**
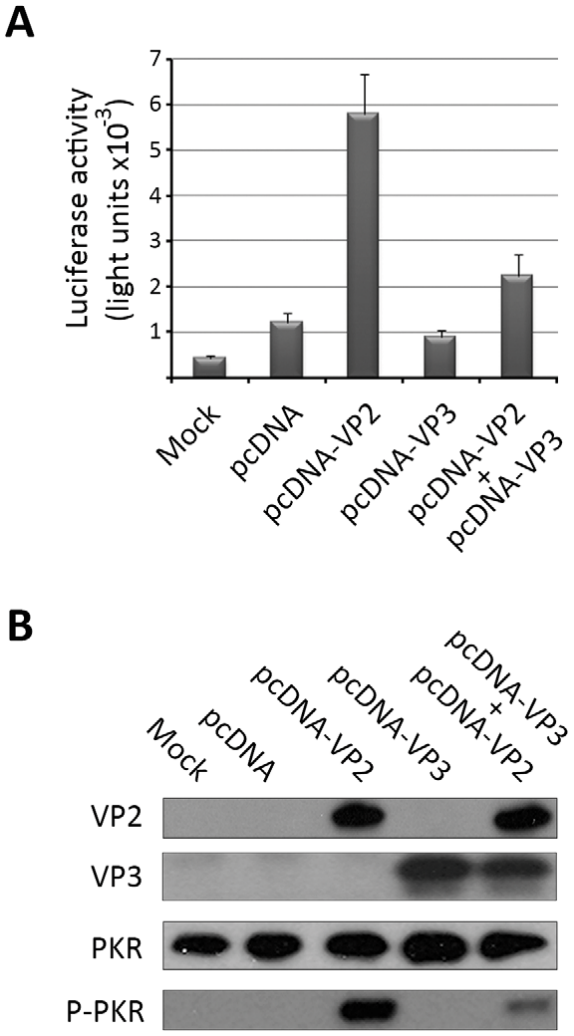
The IBDV VP3 polypeptide counteracts VP2-induced cell responses when expressed from an eukaryotic plasmid expression vector. A. Induction of apoptosis. Cell monolayers were mock-transfected, transfected with pcDNA3, pcDNA-VP2, pcDNA-VP3, or cotransfected with pcDNA-VP2 and pcDNA-VP3. Apoptosis was measured using the Caspase-Glo 3/7 assay kit at 48 h. post-transfection. Each determination was carried in triplicate. Presented data correspond to the mean ± the standard deviation of three independent experiments. **B. PKR phosphorylation.** Total extracts from cells mock-transfected, transfected with pcDNA3, pcDNA-VP2, pcDNA-VP3, or cotransfected with pcDNA-VP2 and pcDNA-VP3 were harvested at 48 h. post-transfection. Extracts were subjected to SDS-PAGE, transferred to nitrocellulose, and immunoblotted with serum anti-VP2, -VP3, -PKR, or -pT451 PKR. The WB corresponding to PKR was used as protein loading control.

We have previously shown that VP2 expression triggers a PCD response when transiently expressed from eukaryotic expression vectors [26]. It was therefore interesting to determine whether the expression of VP3 might also counteract the VP2-mediated PCD response in the absence of exogenous VACV contributions. For this, HeLa cell monolayers were transfected with pcDNA-VP2 or pcDNA-VP3, expressing the VP2 and VP3 polypeptides, respectively or cotransfected with pcDNA-VP2 and pcDNA-VP3. As controls for these experiments, cells were either mock-transfected or transfected with the parental pcDNA3 plasmid. After transfection, cells were maintained for 48 h. and then used to determine the level of caspase 3/7 activation, and the accumulation of relevant polypeptides. As shown in Fig. 4A, whilst cultures transfected with pcDNA3 or pcDNA-VP3 showed caspase activation levels slightly higher than those detected in mock-transfected cells, monolayers transfected with the VP2-expressing plasmid, pcDNA-VP2, exhibited an increase of ca. 10-fold with respect to untransfected control cells. In line with previous observations, VP3 coexpression resulted in a clear reduction on the level of caspase 3/7 activation with respect to those observed in cells overexpressing VP2. These results are in good agreement with the corresponding WB data (Fig. 4B) showing that transfection with pcDNA-VP2 triggers a conspicuous accumulation of P-PKR which is greatly attenuated in cells cotransfected with pcDNA-VP3.

Taken together these results demonstrate that VP3 effectively prevents the PCD response triggered by the VP2 protein regardless of whether the proteins are expressed from VACV- or plasmid-based vectors, and that this effect is directly associated to the blockade of PKR phosphorylation.

### The VP3 dsRNA-binding domain is necessary for its ability to control the VP2-induced PKR activation and PCD response

We have recently mapped the position of a positively charged region located at the surface of the VP3 dimer which is essential for dsRNA binding. This region, termed Patch1, contains four positively charged residues (K99, R102, K105 and K106). We have shown that substitution of positively charged K and R residues for negatively charged D residues (K99D, R102D, K105D and K106D) within the Patch1 region results in the generation of a mutant VP3 version, VP3MutPatch1, lacking the ability to bind dsRNA (unpublished results). We sought to determine whether the capacity of VP3 to counteract the effects triggered by VP2 expression was linked to its ability to bind dsRNA. A recombinant VACV, VT7/VP3P1, was generated and used to perform a comparative analysis of the ability of wild type VP3 and VP3MutPatch1 to prevent cell responses associated to VP2 expression.

**Figure 5:**
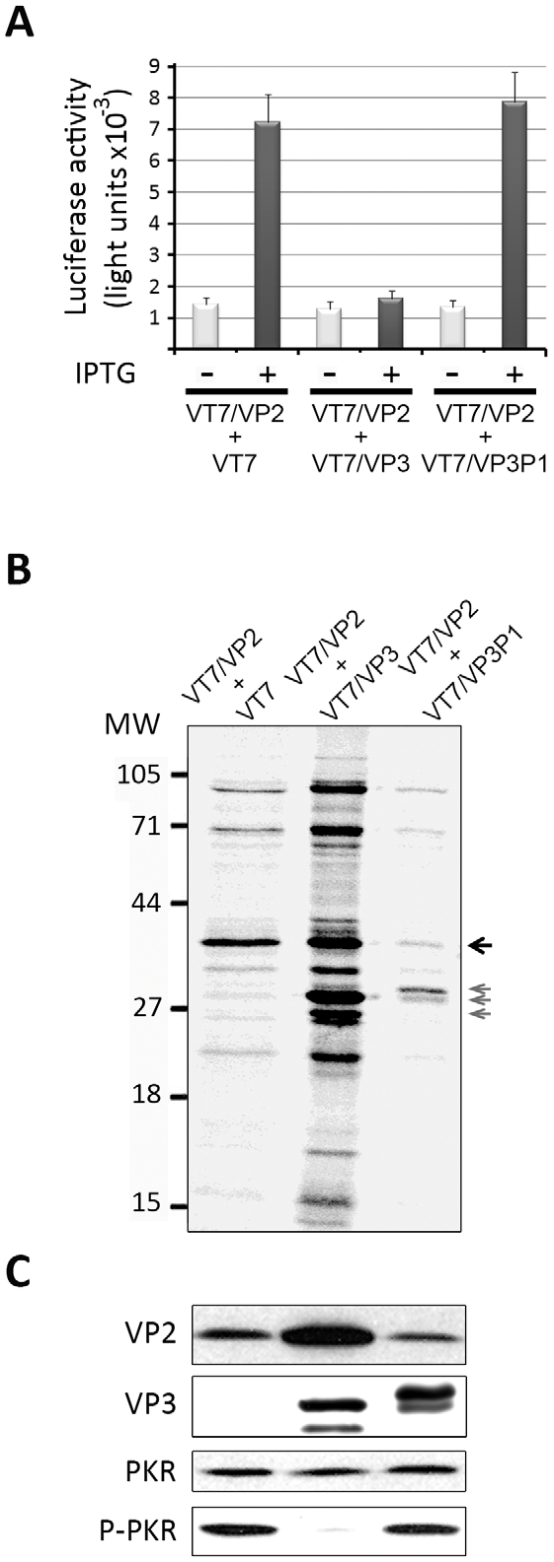
Elimination of the VP3 dsRNA-binding domain abrogates its ability to counteract VP2-induced cell responses. A. Induction of apoptosis. HeLa cell monolayers were infected with VT7/VP2 and coinfected with either VT7, VT7/VP3 or VT7/VP3-P1 (2 PFU/cell of each virus). Cultures were maintained the absence (−) or presence (+) of IPTG. Apoptosis was measured using the Caspase-Glo 3/7 assay kit at 24 h.p.i. Each determination was carried out in triplicate. Presented data correspond to the mean ± the standard deviation of three independent experiments. **B. Host cell protein synthesis.** The panel shows an autoradiography corresponding to the protein synthesis profile obtained from cells infected with VT7/VP2 and coinfected with VT7, VT7/VP3, or VT7/VP3-P1. Cultures were maintained in the presence of IPTG. At 24 h.p.i. cells were metabolically labeled with [^35^S]methionine. Cell extracts were subjected to SDS-PAGE and autoradiography. The position of molecular mass markers is indicated (MW). Arrows indicate the position of bands corresponding to the VP2 polypeptide (black) and VP3 (grey). **C. PKR and eIF2α phosphorylation.** Total cell extracts from IPTG-induced cells infected with VT7/VP2 and coinfected with either VT7, VT7/VP3, or VT7/VP3P1 were subjected to SDS-PAGE, transferred to nitrocellulose, and immunoblotted with serum anti-VP2, -VP3, -PKR, or -pT451 PKR. The WB corresponding to PKR was used as protein loading control.

Three sets of cells were coinfected with VT7/VP2+VT7, VT7/VP2+VT7/VP3, or VT7/VP2+VT7/VP3P1, at a MOI of 2 PFU/cell of each virus, and maintained either in the presence or absence of IPTG. At 24 h.p.i. infected cell cultures were used to assess the induction of PCD, the status of protein synthesis and the presence of VP2, VP3, and total and P-PKR. As shown in Fig. 5A, whilst the expression of the wild type VP3 polypeptide efficiently prevents the activation of caspases 3 and 7 triggered by VP2 expression, the mutant VP3MutPatch1 protein is not able of preventing this effect. In line with this finding, IPTG-induced cells coinfected with VT7/VP2+VT7/VP3P1 showed a blockade of protein synthesis akin to that observed in cells coinfected with VT7/VP2+VT7 (Fig. 5B). The results of the WB analysis confirmed the correct expression of the VP3MutPatch1 polypeptide, and showed that the synthesis of this mutant polypeptide does not prevent the accumulation of P-PKR in cells expressing VP2 (Fig. 5C). As observed in Fig. 5B and C, the VP3MutPatch1 mutant polypeptide exhibits a slightly slower SDS-PAGE migration than its wild type counterpart. These results show that the described anti-apoptotic activity of VP3 is dependent upon the presence of a functional Patch1 dsRNA-binding domain.

### The IBDV VP3 polypeptide rescues the ability of the VACV WRΔE3L deletion mutant to replicate in HeLa cells

It has been shown that the deletion mutant WRΔE3L derived from the VACV WR strain, lacking the E3L gene encoding the E3 polypeptide, is unable to replicate in IFN-competent cell lines including HeLa cells [47]. Interestingly, the insertion of some recombinant genes encoding dsRNA binding proteins from different viruses, e.g. influenza NS1 [45], avian reovirus sigmaA [44], and porcine rotavirus NSP3 [48], into the genome of WRΔE3L leads to the generation of recombinant VACV viruses capable of overcoming the host cell restriction imposed by the lack of the E3L gene.

**Figure 6:**
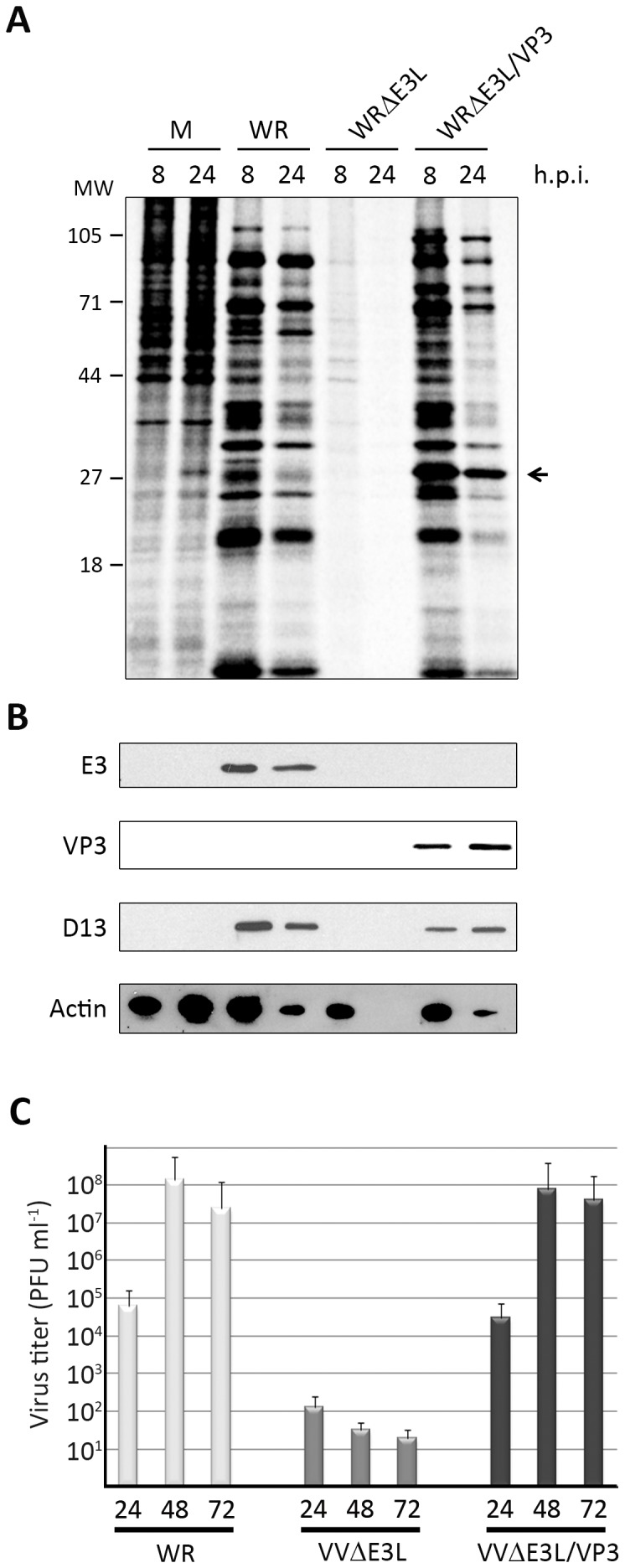
VP3 expression rescues the ability of the WRΔE3L to infect HeLa cells. A. Characterization of protein synthesis. HeLa cell monolayers were mock-infected (M) or infected (MOI 5 PFU/cell) with WR, WRΔE3L, or WRΔE3L/VP3. At the indicated times p.i. (h.p.i.), cells were metabolically labeled with [^35^S]methionine. Cell extracts were subjected to SDS-PAGE and autoradiography. The band corresponding to the VP3 polypeptide is indicated by an arrow. **B. Immunodetection of relevant polypeptides.** Total cell extracts collected at 8 and 24 h.p.i. from mock and infected cultures were subjected to SDS-PAGE, transferred to nitrocellulose, and immunoblotted with serum anti-E3, -VP3, -D13, and -actin. The latter was used as a protein loading control. **C. Growth kinetics.** HeLa cells were infected (0.1 PFU/cell) with WR, WRΔE3L, or WRΔE3L/VP3. At the indicated times p.i. (h.p.i.) cell cultures were harvested and used to determine the virus titers in DF-1 cell monolayers.

Results described above evoked the possibility that the IBDV VP3 polypeptide might functionally replace E3. To test this hypothesis, the VP3 ORF was cloned into the pJR101 VACV insertion/expression plasmid vector under the control of a synthetic early/late VACV promoter. The resulting plasmid, pJR101/VP3, was used generate the recombinant VACV WRΔE3L/VP3 by transfection of WRΔE3L-infected DF1 cells. The DF1 cells are immortalized chicken embryo fibroblasts permissive for WRΔE3L replication. As described in the materials and methods section, the WRΔE3L/VP3 recombinant virus expressing the VP3 polypeptide was generated, selected, grown and titrated in DF-1 cells. In order to characterize the ability of the VP3 polypeptide to overcome the blockade of protein synthesis resulting from the absence of the E3L gene in a non-permissive cell line, HeLa cell cultures were infected (MOI 5 PFU/cell) with WRΔE3L/VP3. As controls for this experiment HeLa cells were also infected with either wild type WR, containing the E3L gene, or with the deletion mutant WRΔE3L. At 8 and 24 h.p.i., cells were either subjected to metabolic labeling with [^35^S]methionine to analyze the protein synthesis rate, or collected for WB analysis. As shown in Fig. 6A, cells infected with WRΔE3L show a clear arrest of protein synthesis that is already detectable at 8 h.p.i. As it has been reported before [31], the shut off of protein synthesis detected in HeLa cells infected WRΔE3L was also associated with the induction of cell death and the subsequent detachment of cells from the plastic culture surface (data not shown). Indeed, the WB carried out with anti-actin serum, used as a protein loading control, revealed that samples collected at 24 h.p.i. from WRΔE3L-infected cultures were completely devoid of protein (Fig. 6B). In contrast, cultures infected with WR or WRΔE3L/VP3 remained translationally active at 24 h.p.i. (Fig. 6A). WB analyses carried out with sera specific for the E3 and the VP3 polypeptides showed that, as expected, the WRΔE3L/VP3 expresses the VP3 polypeptide but is deficient for the E3 protein (Fig. 6B), thus further confirming the genetic identity of this recombinant virus. The VACV D13 polypeptide is encoded by a gene belonging to the late temporal gene class, and was used here as a control of VACV late gene expression [49]. The WB carried out with anti-D13 serum (Fig. 6B) showed the presence of the D13 polypeptide in WRΔE3L/VP3-cells, thus indicating that WRΔE3L/VP3 is capable of completing a replication cycle within a cell system that is non-permissive for the parental WRΔE3L virus. This observation is further supported by the finding that WRΔE3L/VP3 exhibits a plaque size phenotype in HeLa cells akin to that of WR whilst the parental WRΔE3L virus does not induce the formation of lysis plaques in this cell system (data not shown). Finally, the ability of WRΔE3L/VP3 to productively replicate in HeLa cells was also analyzed by infecting these cells at an MOI of 0.1 PFU/cell. Cell samples were harvested at 24, 48 and 72 h.p.i., and used to determine virus titers in DF-1 cells. As an internal control for this experiment, infections and titrations were also carried out with WR and the deletion mutant WRΔE3L viruses. The results of this analysis, shown in Fig. 5C, demonstrate that in contrast to WRΔE3L, unable to grow in HeLa cells, the recombinant WRΔE3L/VP3 efficiently replicates in HeLa cells, rendering virus titers similar to those observed in cells infected with WR. These results demonstrate that IBDV VP3 gene restores the ability of WRΔE3L to productively infect HeLa cells.

## Discussion

To gain further insight into the molecular events leading to the PCD response induced by the expression of the IBDV VP2 gene we have taken advantage of the VACV expression system. This system has been successfully applied to characterize the function of a wide variety of cellular and viral genes involved in apoptosis-related pathways [50]. Results presented here show that the shut off of protein synthesis and the apoptotic response induced by the expression of the IBDV VP2 gene in HeLa cells [26] are preceded by a conspicuous phosphorylation of two host cell polypeptides, namely PKR and eIF2α, that play a critical role in the cellular responses against different types of stress, including those caused by viral infections [38]. Upon activation, PKR undergoes a major conformational rearrangement that leads to its homodimerization and autophosphorylation. Then, PKR dimers interact specifically with the translation initiation factor eIF2α and, in a reaction mediated by the PKR kinase domain, phosphorylate eIF2α at the S51 residue [51]–[53]. eIF2α, together with the initiator Met-tRNAi^Met^ and GTP, participates in the selection of the translation start codon [54]–[55]. Phosphorylation of eIF2α S51 blocks the exchange of eIF2-GDP to eIF2-GTP, thus abrogating translational initiation [56]. In addition to its role in the inhibition of protein synthesis, it has been reported that the presence of activated PKR increases the expression of several proapoptotic genes, including Fas and Bax [57]. PKR also induces apoptosis by other mechanisms entailing the activation of Fas-associated death domain/caspase 8 [57], p53 phosphorylation [58], and by modulating the activity of the nuclear factor κB (NF-κB) [59], [60].

Our results show that the simultaneous expression of a non-phosphorylatable dominant negative mutant form of the PKR polypeptide together with VP2 prevents the activation of PKR and the subsequent eIF2α phosphorylation, and thus thwarts the ensuing arrest of protein synthesis and PCD response in HeLa cells. Indeed, taken together, our results indicate that VP2 expression activates a PKR-mediated PCD response.

PKR is activated either by binding to dsRNA or through the interaction with different polypeptides [61]. It is difficult at this point to discriminate whether the activation of PKR observed in cells expressing VP2 is prompted by the VP2 polypeptide itself or by the accumulation of VP2 mRNAs. Taking into account results concerning the antiapoptotic activity of the RNA binding VP3 polypeptide discussed below, we favor the hypothesis that the activation of PKR observed in cells expressing VP2 is due to the accumulation of VP2 mRNAs.

We have previously shown that the proapoptotic effect associated to VP2 expression is not a direct consequence of the use of the VACV expression system (VACV VOTE) [32]. Indeed, a similar effect was observed in cells transfected with pcDNA-VP2, a derivative of the eukaryotic expression plasmid vector pcDNA3, in which the transcription of the VP2 ORF, under the control of the immediate-early promoter-enhancer region of the human cytomegalovirus (CMV), is driven by the host's RNA polymerase II [26]. Additionally, it has been documented that expression of other viral and cellular genes using the same VACV system, including that encoding the IBDV VP3 polypeptide described here, does not induce neither protein synthesis arrest nor PCD response in infected cells [26]. These previous results, confirmed in this report, rule out the hypothesis that VP2 might somehow sabotage the function of the VACV-encoded E3 polypeptide.

Imperfect RNA duplexes of sufficient double stranded character are capable to activate PKR [62], thus it has been shown that extended duplex regions within the 3′ untranslated regions of mRNAs from some cytoeskeletal proteins, i.e. tropomyosin, troponin and cardiac actin, interact with and activate PKR [63]. RNA fold predictions indicate the presence of large stretches of duplex in VP2 mRNAs. Accordingly, it seems feasible that the accumulation of VP2 mRNAs might be sufficient to trigger PKR activation.

The remarkable transcriptional efficiency of the VACV VOTE expression system makes it likely that the amount of VP2 mRNAs produced in cells infected with VT7/VP2 might outnumber available dsRNA-binding VACV E3 molecules, thus allowing the presence of enough “free” VP2 mRNAs to trigger PKR activation. Our results showing that, unlike the wild type VP3, the VP3MutPatch1 polypeptide lacking the ability to bind dsRNA is unable to prevent the apoptotic effect associated to VP2 expression provides an indirect support to this hypothesis. Indeed, the precise mechanism by which VP2 expression triggers PKR phosphorylation deserves an in depth characterization. VP3 is the second major structural IBDV protein [64]. This polypeptide is released simultaneously with pVP2 and the VP4 protease following the autocatalytic processing of the IBDV polyprotein [18]. VP3 is a multifunctional polypeptide that acts as a scaffold during capsid assembly [65], recruits and activates the virus-encoded RdRp VP1 [66], [67], and binds the dsRNA viral genome to build up the ribonucleoprotein (RNP) complexes that occupy the inner space of IBDV particles [13]. The results presented here show that VP3 efficiently precludes the protein synthesis arrest and the PCD response triggered by VP2 expression by inhibiting the activation of PKR and therein eIF2α phosphorylation, and the activation of the apoptotic signaling cascade. The mechanism(s) by which VP3 prevents the VP2-induced activation of PKR remains to be elucidated. It has been shown that the VP3 only interacts with the C-terminal domain of the pVP2 precursor and not with the mature VP2 polypeptide [68]. This rules out a possible mechanism based upon the sequestration of the VP2 polypeptide via a direct VP2/VP3 interaction. Another possibility that we cannot discard at this point is that VP3 might prevent the PCD response via a direct or indirect interaction with the PKR polypeptide. In this regard, direct interaction with PKR of two well characterized proteins, VACV E3 and Influenza NS1, with antiapoptotic properties has been described [69], [70]. This possibility will be investigated. Nonetheless, the ability of VP3 to bind both single stranded (ss)RNA, including a synthetic RNA produced by T7 polymerase transcription corresponding to the IBDV polyprotein ORF, and purified IBDV dsRNA genomic segments [46] and short (21 bp) dsRNA duplexes (unpublished results) suggests that the mechanism used by VP3 to control VP2-mediated PKR activation might involve the binding to VP2 mRNAs duplex regions, thus preventing their recognition by the PKR polypeptide. This hypothesis is strengthened by the finding that the expression of a mutant VP3 unable to bind dsRNA fails to prevent the phosphorylation of PKR induced by VP2 expression. Provided this hypothesis is correct, the presence of VP3 in both IBDV infected cells as well as in cells expressing the IBDV polyprotein might counteract the PKR-activating effect of mRNAs containing the VP2 coding region, thus precluding their proapoptotic effect.

It has been described that the VP3 polypeptide encoded by the infectious pancreatic necrosis virus (IPNV), the prototype member of the *Birnaviridae* family, induces apoptosis via the Bad-mediated mitochondria pathway in fish and mouse cells [71]–[73]. These observations strongly contrast with results described here showing the antiapoptotic role of its IBDV counterpart. The molecular basis underlying the differential behavior of the IBDV and IPNV VP3 polypeptides are at this point unknown and deserve a detailed analysis.

Data presented here conclusively show that the VP3 protein successfully replaces the VACV E3 polypeptide, restoring the ability of the VACV WRΔE3L deletion mutant to replicate in the non-permissive HeLa cell line. Although a comparative analysis of the E3 and VP3 sequences did not reveal significant domain similarities amongst them (data not shown), the described data suggests that VP3 might play a role similar to E3 in IBDV-infected cells counteracting the host's innate antiviral response.

Birnaviruses exhibit some structural features that strongly differentiate them from all other icosahedral dsRNA viruses [74]. Prototypal dsRNA viruses, e.g. reoviruses, possess two concentric capsids, and their dsRNA genomes remain permanently enclosed within the innermost capsid, known as T = 2 core [75]. This structure provides the enzymatic machinery for the synthesis and extrusion of virus mRNAs, and shelters the dsRNA genome from cellular dsRNA sensors [76]. In contrast, birnaviruses posses a single capsid that encloses RNP complexes formed by the dsRNA segments covalently linked to the VPg form of the RdRp and wrapped up by VP3. At this point it is not known whether birnavirus particles are disassembled during the entry process. However, we have shown that purified IBDV RNPs are transcriptionally active [13]. This suggests the possibility that RNPs might be released and act as capsid-independent transcription complexes. In this putative scenario, the VP3 polypeptide might play a key role shielding the dsRNA against cellular dsRNA sensors and thus preventing the onset of dsRNA-mediated innate immune responses.
